# Optimization of the *piggyBac* Transposon Using mRNA and Insulators: Toward a More Reliable Gene Delivery System

**DOI:** 10.1371/journal.pone.0082559

**Published:** 2013-12-03

**Authors:** Solenne Bire, Déborah Ley, Sophie Casteret, Nicolas Mermod, Yves Bigot, Florence Rouleux-Bonnin

**Affiliations:** 1 GICC, UMR CNRS 7292, Université François Rabelais, Tours, France; 2 Institute of Biotechnology, University of Lausanne, and Center for Biotechnology UNIL-EPFL, Lausanne, Switzerland; 3 PRC, UMR INRA-CNRS 7247, Centre INRA Val de Loire, Nouzilly, France; Southern Illinois University School of Medicine, United States of America

## Abstract

Integrating and expressing stably a transgene into the cellular genome remain major challenges for gene-based therapies and for bioproduction purposes. While transposon vectors mediate efficient transgene integration, expression may be limited by epigenetic silencing, and persistent transposase expression may mediate multiple transposition cycles. Here, we evaluated the delivery of the *piggyBac* transposase messenger RNA combined with genetically insulated transposons to isolate the transgene from neighboring regulatory elements and stabilize expression. A comparison of *piggyBac* transposase expression from messenger RNA and DNA vectors was carried out in terms of expression levels, transposition efficiency, transgene expression and genotoxic effects, in order to calibrate and secure the transposition-based delivery system. Messenger RNA reduced the persistence of the transposase to a narrow window, thus decreasing side effects such as superfluous genomic DNA cleavage. Both the CTF/NF1 and the D4Z4 insulators were found to mediate more efficient expression from a few transposition events. We conclude that the use of engineered *piggyBac* transposase mRNA and insulated transposons offer promising ways of improving the quality of the integration process and sustaining the expression of transposon vectors.

## Introduction

For the purposes of gene therapy or bioproduction, stable and long-lasting transgene expression is usually required. In such case, stable integration of the transgene in the host genome is often necessary. This may be achieved using integrative viral or non-viral plasmid vectors. However, virus-based vectors are immunogenic, they can cause genotoxic effects, their cargo capacity is limited, and the large-scale production of viral particles is complex [1]. DNA transposons appear as a promising non-viral alternative: they have a greater cargo capacity while maintaining highly efficient transgene integration [2], and they are considered to be less immunogenic than viral vectors [3]. Native DNA transposons are mobile genetic elements encoding a single enzyme, the transposase, which is able to recognize transposon-specific inverted terminal repeats (ITRs) situated at both ends of the transposon. Some transposases act to excise and reintegrate the transposon at other sites in the genome by a “cut-and-paste” mechanism. By replacing the transposase gene with a gene of interest between the two ITRs, and by supplementing the transposase source *in trans*, transposons have become useful integrating vectors to reshape the genome of various cells lines and organisms [4].

Among the eukaryotic transposons, *Sleeping Beauty (SB*) has been the first shown to be active in human and mouse cells [5,6]. *SB* has also been involved in preclinical trials [7], in the perspective of human clinical trials. The *piggyBac* transposon (PB), originally isolated from insect cells, was recently added to the transposon toolbox for mammalian cell engineering [8-10]. This element has the highest transposition rates and cargo capacities in mammalian cell lines [11], and the basic vector is relatively free from intellectual property restrictions [12]. *PB* has been used for transgenesis in mice [13], for gene transfer in human cells [14], and to reprogram fibroblasts into induced pluripotent stem cells [15]. Moreover, *PB* excises precisely upon transposition and would be less susceptible to transposase overproduction inhibition or local hopping than other transposons [13]. Optimized *PB* transposon systems with more active transposases are also available [16,17]. Finally, genome-wide integration analysis indicated that *PB* has a slight bias towards integrating in cellular gene or promoter sequences when compared to *SB* [18], suggesting an increased risk of insertional genotoxicity for the former transposon systems.

Transposition usually relies on the co-transfection of a donor plasmid carrying the gene of interest and a helper plasmid carrying the transposase gene under the control of a strong promoter. The main drawback of this approach is the lasting presence of the transposase due to the persistence of the episomal plasmid DNA (pDNA). This in turn may lead to multiple transposition cycles and thus increase potential damages to the chromosome upon transposon excision and chromosome break repair by cellular end-joining and recombination activities. A more labile source of transposase, such as messenger RNA (mRNA), should offer a more suitable alternative to narrow the transposase expression and to achieve more stable genomic integrations of the transgene, while decreasing potential mutagenic effects [19]. Efficient transposition using mRNA-encoded transposases has been documented in various species including mammals [20,21]. 

Once integrated into a chromosome, transgenes may undergo silencing epigenetic effects [22]. One way to circumvent transgene extinction is to integrate chromatin-control elements, such as insulators, into the transposon cassette. Insulators are DNA *cis*-regulatory elements having chromatin boundary and/or enhancer-blocker properties [23]. Boundary insulators prevent the propagation of a silencing chromatin structure over the transgene, thus mediating higher and/or more persistent levels of transgene expression. Enhancer-blocker insulators specifically prevent the insertional activation of adjacent cellular genes by the regulatory elements of the vector and conversely. A lot of these sequences are subjected to an extensive patenting since their discovery [24]. At present, several insulator sequences have been assessed in various cellular or vector systems, including the cHS4, D4Z4, CTCF and CTF/NF1 insulators [25-28]. However, a systematic comparison of their potency is currently not available.

Using the *piggyBac* model, the aim of this study was to improve the reliability and stability of gene transfer by the *PB* transposon by delivering the source of transposase as an mRNA and by integrating insulator elements in the transposon cassette. We reduce the window time during which the transposase is present in the cells and show that high transposition efficiency can be achieved without detectable genotoxicity. We identify insulating sequences that improve expression of the transgene from just a few transposed vector copies in human cells. Overall, we conclude that modulating *PB* transposase bioavailability using exogenous mRNA and insulating transposon-born transgenes offer a promising approach to achieve both more secure transgene integration as well as higher and more stable transgene expression.

## Materials and Methods

### Plasmid constructs

#### Helper plasmid

The V5PB pDNA (6.4 kbp) encodes the V5 tagged *PB* transposase surrounded with the 5’ and 3’ untranslated terminal regions of the *Xenopus laevis* β-globin gene and inserted into the pCS2+ vector (Life Technologies, Paisley, UK). The V5PB sequence (2067 bp) did not contain any AU-rich elements, 5’ stem loops, premature translation termination codons or introns (GenBank accession number: EF587698), and was synthesized by ATG:biosynthetic, Merzhausen, Germany. 

#### Donor plasmids

pBSK ITR5'-Neo^R^-ITR3' (5.2 kbp) was built by introducing the *PB* 3’ and 5’ inverted terminal repeats (ITRs) (http://piggybac.bio.nd.edu/GenBank%20Format/pBSII-ITR1.1k) into the pBluescript SK plasmid. The neomycin phosphotransferase gene (Neo^R^, 1.5 kbp) was cloned between the two ITRs. Two copies of the 1.2 kbp chicken ß-globin HS4 insulator (2xcHS4 2.4 kbp), one copy of the human core D4Z4 insulator (65 bp), six copies of the 40 bp CTCF binding site (6xCTCF, 240 bp) or seven copies of the 20 bp CTF/NF1 binding site (7xCTF/NF1, 140 bp), were inserted on either side of the transgene. The D4Z4 sequence was synthesized according to patent application WO2009/016206. 

#### Control plasmid

The GFP pDNA (5.1 kbp) vector was built by ligating the green fluorescent protein gene in the pCS2+. It was used as a negative control of transposition (no transposase).

### *In vitro* mRNA synthesis

V5PB or GFP pDNA template preparation and *in vitro* transcription was performed according to the Manufacturer’s instructions using the MEGA Script SP6 kit combined with the ARCA (Anti-Reverse Cap Analog) kit and polyadenylated using the Poly(A) Tailing kit (Life Technologies). V5PB mRNA (2.5 kb) and GFP mRNA (1.3 kb) were checked by 0.8% agarose gel electrophoresis (Figure S1) and the concentration was determined by measuring the absorbance at 260 nm.

### Cell culture and nucleic acid transfection

HeLa cells (ATCC CCL-2) were cultured in DMEM-10% heat-inactivated fetal bovine serum (PAA Laboratories, Pasching, Austria) at 37°C and 5% CO_2_-containing atmosphere. The cells were plated at a density of 1.10^5^ cells per well in 24-well plates, and grown for 24 h to 80% confluence. All transfections were performed using jetPEI™ (Polyplus Transfection, Illkirch, France) at an N/P ratio of 5 according to the Manufacturer's instructions. When mRNA and pDNA were co-transfected, complexes were formed separately.

### Western-Blot

For the quantitative studies, cells were transfected with 0 to 500 ng of transposase encoding mRNA or pDNA and extracted 24 h post-transfection. For the kinetics of expression study, cells were transfected with 187.5 ng of V5PB mRNA or pDNA, and extracted at various times. Transfected cells were harvested with extraction buffer (0.5% SDS, 100-mM NaCl, 10 mM ß-mercaptoethanol and 1X protease inhibitor). 15 µg of protein extract were loaded per lane on a 10% SDS–PAGE gel, then transferred to a Hybond-ECL^TM^ (GE Healthcare, Little Chalfont, UK) membrane blocked with 5% non-fat dry milk dissolved in PBS-Tween^®^20 0.05%. *PB* transposase (termed V5PB Tp), Menin, β-actin or phosphorylated H2AX detection was performed using specific antibodies dissolved in blocking solution (Table S1) and using an ECL^TM^ Western-Blot Analysis System (GE Healthcare) and a LAS-4000 apparatus (Fujifilm, Tokyo, Japan). Proteins were quantified using MultiGauge 4.0 software and normalized to an endogenous protein (Menin or β-actin). Experiments were performed in triplicate.

### Protein and nucleic half-life assays

#### PB mRNA

Cells were transfected with 187.5 ng of V5PB mRNA and total cellular RNA was extracted at different times using the Nucleospin RNA II kit (Macherey-Nagel, Dueren, Germany), reverse transcribed using the RevertAid First Strand cDNA Synthesis kit (Roche Applied Science, Penzberg, Germany), and subjected to real-time quantitative analysis on a MiniOpticon Real-Time PCR System instrument and software (Bio-Rad, Hercules CA, USA) with MesaGreen qPCR Master Mix Plus for SYBR Assay NoRox kit (Eurogentec, Seraing, Belgium). Reactions were performed in triplicate with specific primers amplifying V5PB mRNA or 18S RNA (Table S2). 

#### PB pDNA

Cells were transfected with 187.5 ng of V5PB pDNA and plasmid rescue assay was performed at different times [29]. 

#### PB protein

Cells were transfected with 187.5 ng of V5PB transposase mRNA, incubated 18 h and then treated with 30 µg/mL of cycloheximide to shut down protein expression. The decrease in protein was investigated by Western-Blot analysis at different times.

### Transposition assay

Cells were co-transfected with 0 to 500 ng of V5PB transposase pDNA or mRNA and with equal amounts of donor plasmid (1:1 ratio). Two days after transfection, cells were transferred in 100-mm plates and selected with G418 sulfate (800 µg/mL, PAA Laboratories) for 15 days. Cells were fixed and stained with 70% EtOH-0.5% methylene blue and colonies >0.5 mm in diameter were counted. To consider only integration events due to transposition, the number of colonies observed in the presence of the transposase source was subtracted by the number of colonies obtained in the respective negative control performed without transposase. Experiments were performed in triplicate.

### Copy number of genome integrated transgene or V5PB transposase gene

After transposition assay, total DNA was isolated from stable cell pools using the DNeasy Tissue Kit (Qiagen, Hilden, Germany) according to the Manufacturer’s protocol. 10 ng of genomic DNA were analyzed by quantitative PCR (LightCycler1 480 real-time PCR system, Roche Applied Science) using the SYBR Green-Taq polymerase kit (Eurogentec) to amplify the Neo^R^, V5PB and GAPDH genes (Table S2). The ratios of the Neo^R^ and V5PB gene copy numbers were calculated relative to that of the reference gene GAPDH in the human genome. Reactions were performed in triplicate.

### Statistical analysis

Values are the mean ± standard deviation of experiments done in triplicate. Shapiro-Wilk test confirmed the normality of each set of samples. The significance of the differences between groups with the control was tested using the Kruskal-Wallis test with p<0.05.

## Results

### PB transposase expression from mRNA transfection

**Figure 1:**
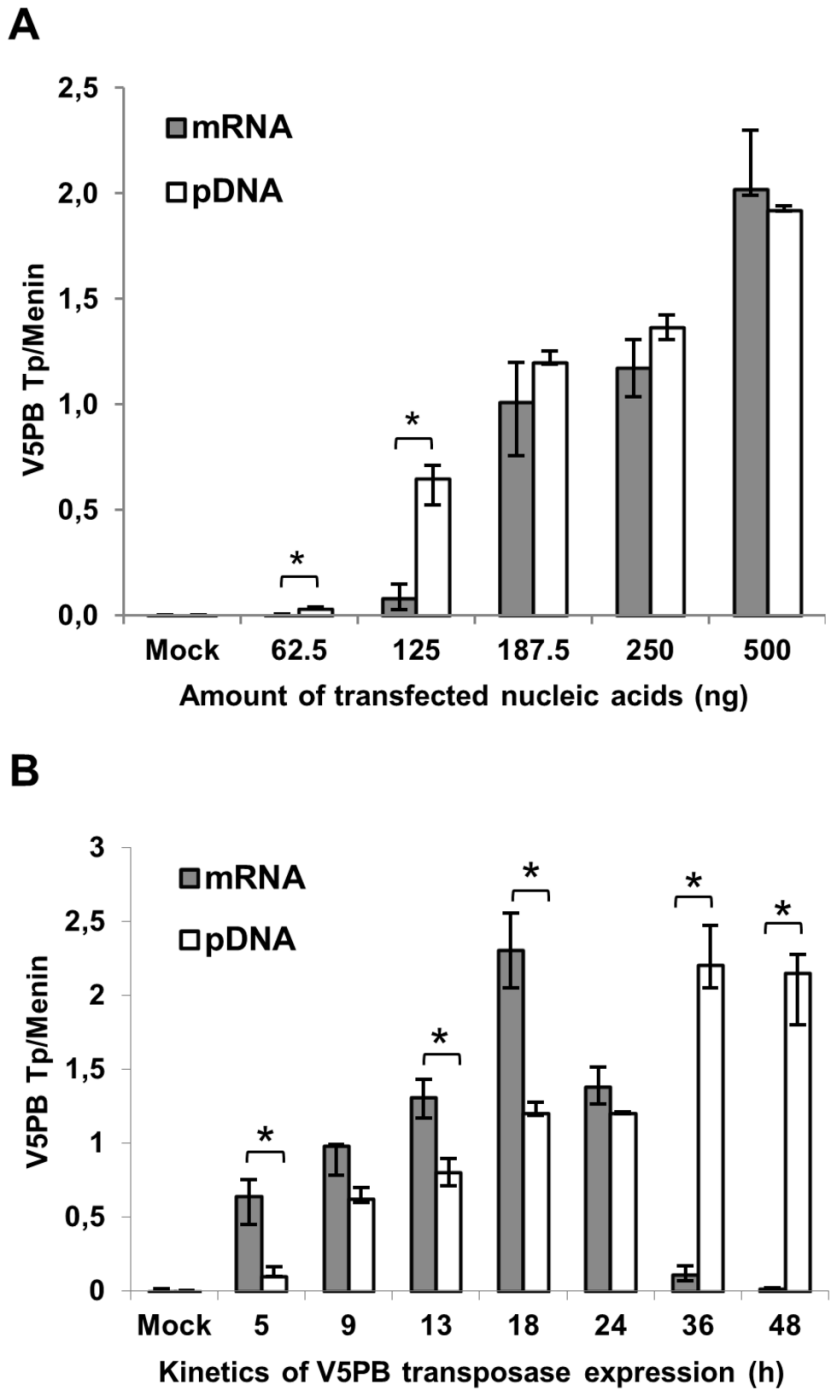
Expression (A) and kinetic (B) of *PB* transposase by type and quantity of nucleic acid. HeLa cells were transfected with indicated amounts (A) or 187.5 ng (B) of *PB* mRNA or pDNA and total protein extraction was performed 24 h (A) or at indicated times (B) post-transfection. Transposase (V5PB Tp) expression was determined by Western-Blot and protein quantification was normalized to the endogenous Menin protein. Values represent the average of 3 experiments done in triplicate. Mock: untransfected cells. * Indicates statistically significant differences between mRNA and pDNA (p<0.05).

We first compared transposase expression in terms of protein quantity and persistence from each type of nucleic acid vector. The mRNA and pDNA vectors yielded dose-dependent transposase expression levels during the first 24 h post-transfection, reaching comparable maximal amounts in the range tested (Figure 1A). However, the smaller amounts of mRNA led to significantly less transposase than an equivalent amount of pDNA (8-fold for 62.5 ng and 125 ng, p<0.05). Therefore, the transposase expression levels could be modulated by the quantity and type of nucleic acid transfected in the cells, and high levels of expression could be reached by transfecting high mRNA amounts, despite its lower efficacy on a molar basis. In following experiments, 187.5 ng of mRNA or pDNA was used to transfect cells, as these amounts yielded comparable transposase levels. The kinetic of transposase expression showed distinct patterns depending on the nucleic acid transfected (Figure 1B). The pDNA led to little transposase expression 5 h post-transfection, followed by a gradual rise to reach a 10-fold increase 36 h post-transfection and a plateauing effect at 48 h. Using mRNA, significant transposase expression was observed after 5 h, further rising to reach a peak at 18 h. Thereafter, transposase levels quickly declined to background levels and only traces were detected at 48 h. Thus, protein synthesis from an mRNA source was both faster and more transitory than that obtained from the pDNA, as may be expected.

**Figure 2:**
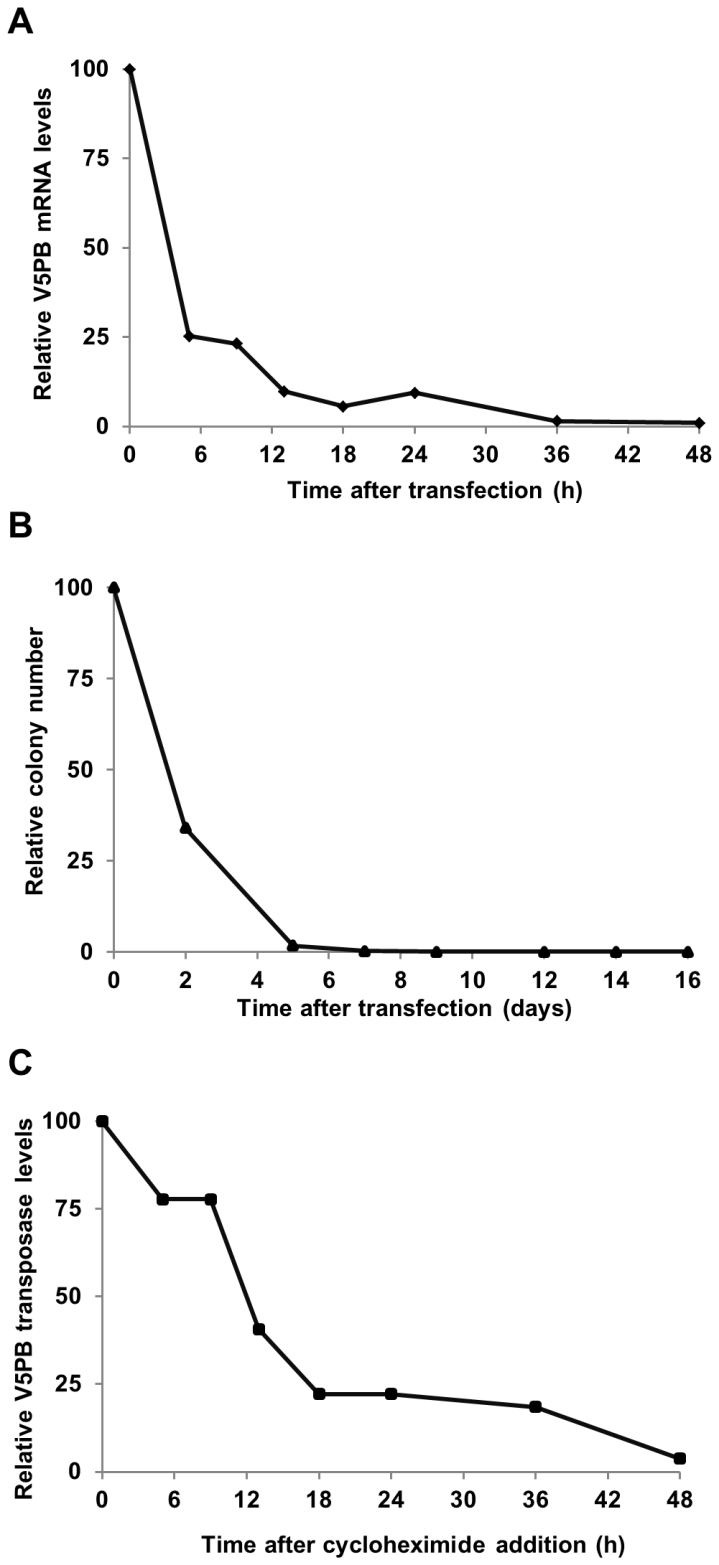
*piggyBac* transposase bioavailability. (**A**) **Half-life of the *PB* mRNA**. Cells were transfected with 187.5 ng of *PB* mRNA. Total RNA was extracted at indicated times, reverse transcribed and subjected to qPCR. 18S RNA served as an internal standard to normalize the data. (**B**) **Persistence of *PB* pDNA after transfection**. Cells were transfected with 187.5 ng of *PB* pDNA and plasmid rescue was performed at 0 to 20 days. Ampicillin-resistant colonies were selected to evaluate the persistence of the plasmid. (**C**) **Half-life of the *PB* transposase (V5PB Tp)**. Cells were transfected with 187.5 ng of *PB* mRNA, incubated 18 h to reach the peak of transposase expression and treated with cycloheximide (t0=100). Total protein extraction was done at the indicated times from t0. The transposase half-life was determined by Western-Blot and quantification was normalized to the endogenous actin protein.

Correspondingly, the half-life of the *PB* mRNA was estimated to be ~3 h (Figure 2A). Only traces of mRNA were detected 36 h post-transfection, which is consistent with the decrease in protein levels observed in Figure 1. The loss of pDNA following transfection was much slower than that of the mRNA, as the pDNA half-life was ~36 h and as it was still detectable 5 days post-transfection (Figure 2B). The transposase bioavailability was studied by determining the half-life of the protein. To do so, cells were transfected with the *PB* mRNA and treated with cycloheximide at the peak of transposase expression (18 h). The decrease in transposase levels post-treatment showed a half-life of ~12 h (Figure 2C), which is consistent with the sharp decline of the transposase expressed from the mRNA vector and from the short half-life of mRNA itself (Figure 1B and 2A). Overall, the mRNA provided a more restricted transposase expression in terms of both quantity and duration.

### PB mRNA-based transposition in mammalian cells

**Figure 3:**
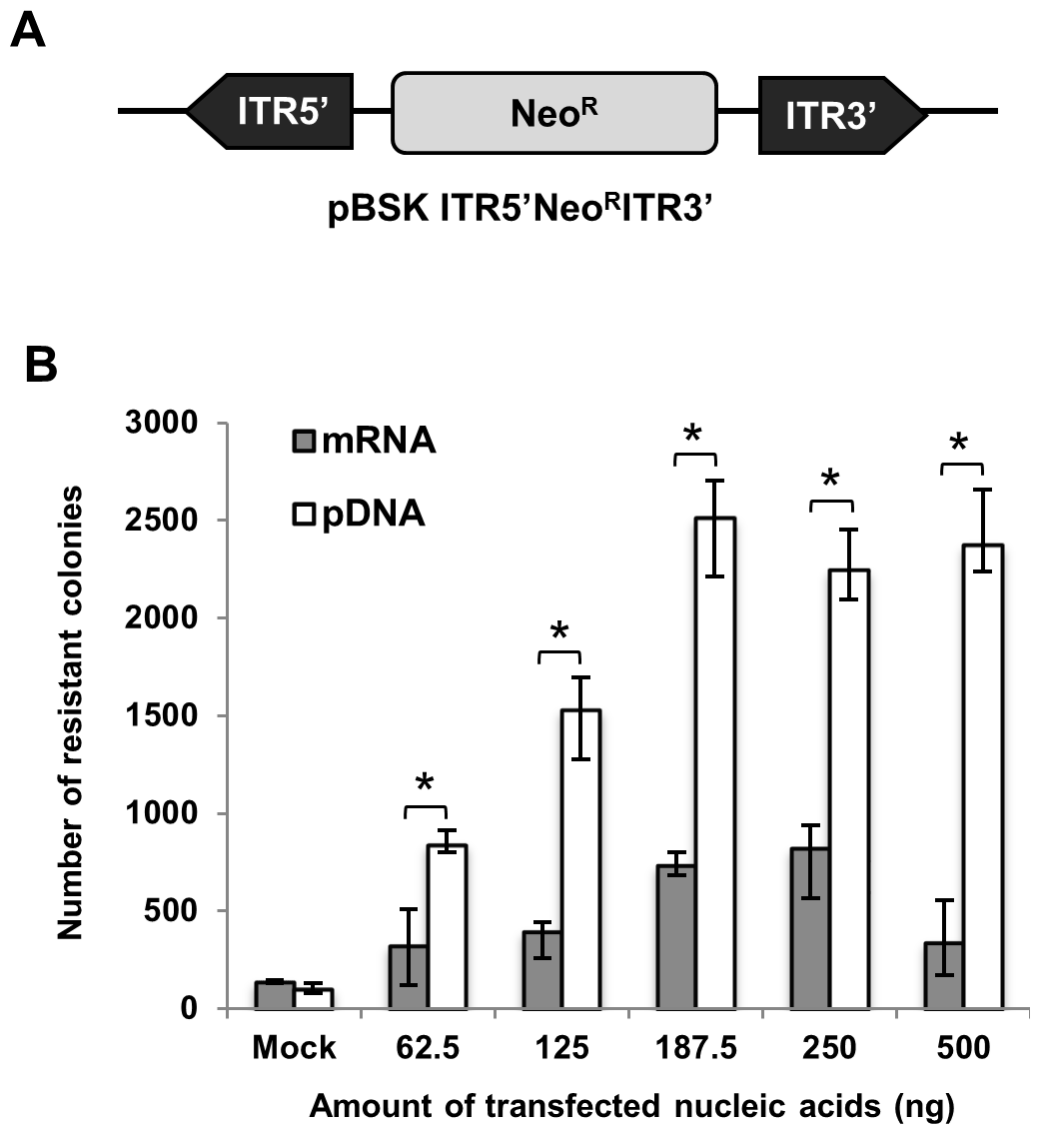
Transposition assay in mammalian cells. (A) Donor plasmid harboring the transposable element. This vector is composed of the neomycin phosphotransferase gene (Neo^R^) flanked by the *PB* ITRs. (B) Transposition efficiency by type and quantity of nucleic acid. Cells were transfected with indicated amounts of *PB* mRNA or pDNA alongside with 187.5 ng of donor plasmid. GFP mRNA or pDNA (500 ng) served as negative controls of transposition (Mock: recombination events). To consider only transposition events, the number of colonies observed in the presence of the transposase source was subtracted by the number of colonies obtained in the respective negative control performed without transposase for each quantity. Values represent the average of 3 experiments done in triplicate. * Represents statistically significant difference between mRNA and pDNA (p<0.05).

The functionality and efficiency of the transposase mRNA source was assessed by performing transposition assays using 0 to 500 ng of transposase mRNA or pDNA vectors alongside a fixed quantity of a circular donor plasmid (Figure 3A). In presence of the transposase, a higher number of neomycin-resistant stable colonies were scored using either the mRNA or pDNA when compared to the negative control, indicating that transposition had occurred in the cells (Figure 3B). The *PB* transposase mRNA transfection also yielded a high number of transposition events in another human cell line as well as in CHO cells (Figure S2). The maximal number of transposition events was obtained with 187.5 ng of pDNA and with 187.5 and 250 ng of mRNA, thus correlating well with the protein expression profile (Figures 3 and 1). However, 3 to 5-fold fewer colonies were scored using the mRNA (p<0.05). Quantification of the integrated transgene copies revealed a mean number of ~2 transposable vectors per genome when using either the transposase pDNA or the mRNA (Table 1, top line), implying that the smaller colony number was not due to an intrinsically lower transposition efficiency of the mRNA-expressed transposase, and that it more likely resulted from the short duration of expression. Indeed, transposition events probably occurred mostly during the first 18 h after mRNA transfection, whereas the pDNA-based transposition is expected to persist for longer durations, thus increasing the likelihood of obtaining at least one transposition event in a given cell. No integration of the *PB* transposase gene was detected following mRNA transfection, whereas pDNA transfection led to ~1.5 transgene copies per cell, as resulting from the spontaneous recombination events of one or several plasmids within a genomic DNA locus (Table 1, bottom line). Therefore, the use of mRNA transposase appears more reliable than the pDNA approach to prevent potential secondary transposition cycles.

**Table 1:** Number of transgene or *piggyBac* gene integrations in pools of colonies in the presence of a pDNA or mRNA source of the V5PB transposase.

	V5PB pDNA	V5PB mRNA
Mean number of transposon integrations in pools of colonies	2.2	1.9
Mean number of transposase gene integrations in pools of colonies	1.5	0

### Impact of transposase expression and transposition on cell viability

**Figure 4:**
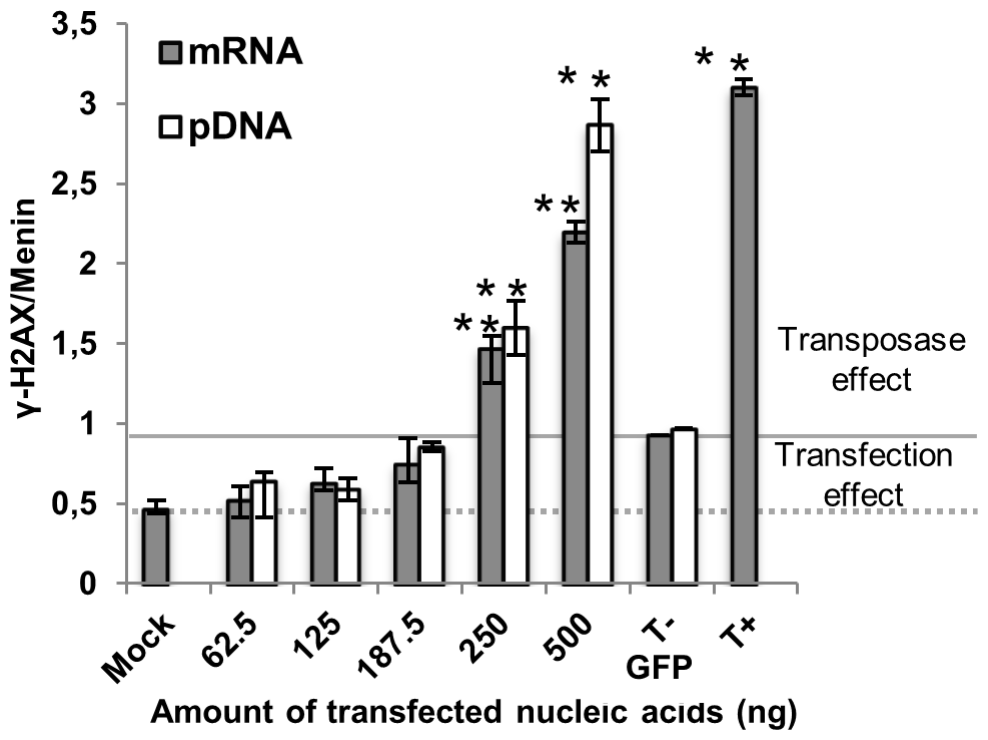
Detection of H2AX phosphorylation following dose-dependent *PB* mRNA or pDNA transfection. Cells were transfected with indicated amounts of *PB* mRNA or pDNA and total protein extraction was performed 24 h post-transfection. γ-H2AX expression was determined by Western-Blot and protein quantification was normalized to the endogenous Menin protein. Mock: untransfected cells. T-_GFP_: cells transfected with 500 ng of GFP mRNA or pDNA. T+: untransfected cells treated with 2 µg/mL doxorubicin (positive control). * Indicates statistically significant differences between treated and untreated cells (p<0.05). Values represent the average of 3 experiments done in triplicate. The signal between the dotted line (mock control) and solid line (T-_GFP_ control) is considered to be due to the “transfection effect”. Above the solid line, the signal is due to the “transposase effect”.

Transposases are known to allow specific DNA double-strand breaks (DSBs) for excision and integration of the transposon DNA. They may also mediate non-specific DSBs at cryptic sites in the genome, which may lead to genotoxicity and genomic damage [17]. We assayed the genotoxicity of the transposase by transfecting varying amounts of *PB* mRNA or pDNA and detecting phosphorylated H2AX (γ-H2AX) as a marker of DSB DNA damages (Figure 4). Untransfected cells (Mock) displayed a basal γ-H2AX signal due to constitutive DSBs occurring spontaneously in the cultured cells (dotted line). The T-_GFP_ control showed a slight increase in the γ-H2AX signal (dashed line), due to DSBs-like structures on the transfected nucleic acids. mRNA and pDNA led to a similar γ-H2AX pattern since comparable amounts of transposase were produced at the DSB quantification time (24 h, Figure 1). No statistical difference in the γ-H2AX signal was observed using ≤187.5 ng of *PB* mRNA or pDNA whereas higher quantities induced γ-H2AX increase comparable to the doxorubicin-treated positive control. So, the amount of transposase produced from ≤187.5 ng of mRNA or pDNA does not result in detectable cytotoxic effects. This result was confirmed using a cell metabolism and proliferation assay (Figure S3), showing that neither transfection nor the presence of the transposase and resulting transposition events affected cell viability under these conditions.

### Use of insulator sequences to isolate the transgene from the surrounding environment

**Figure 5:**
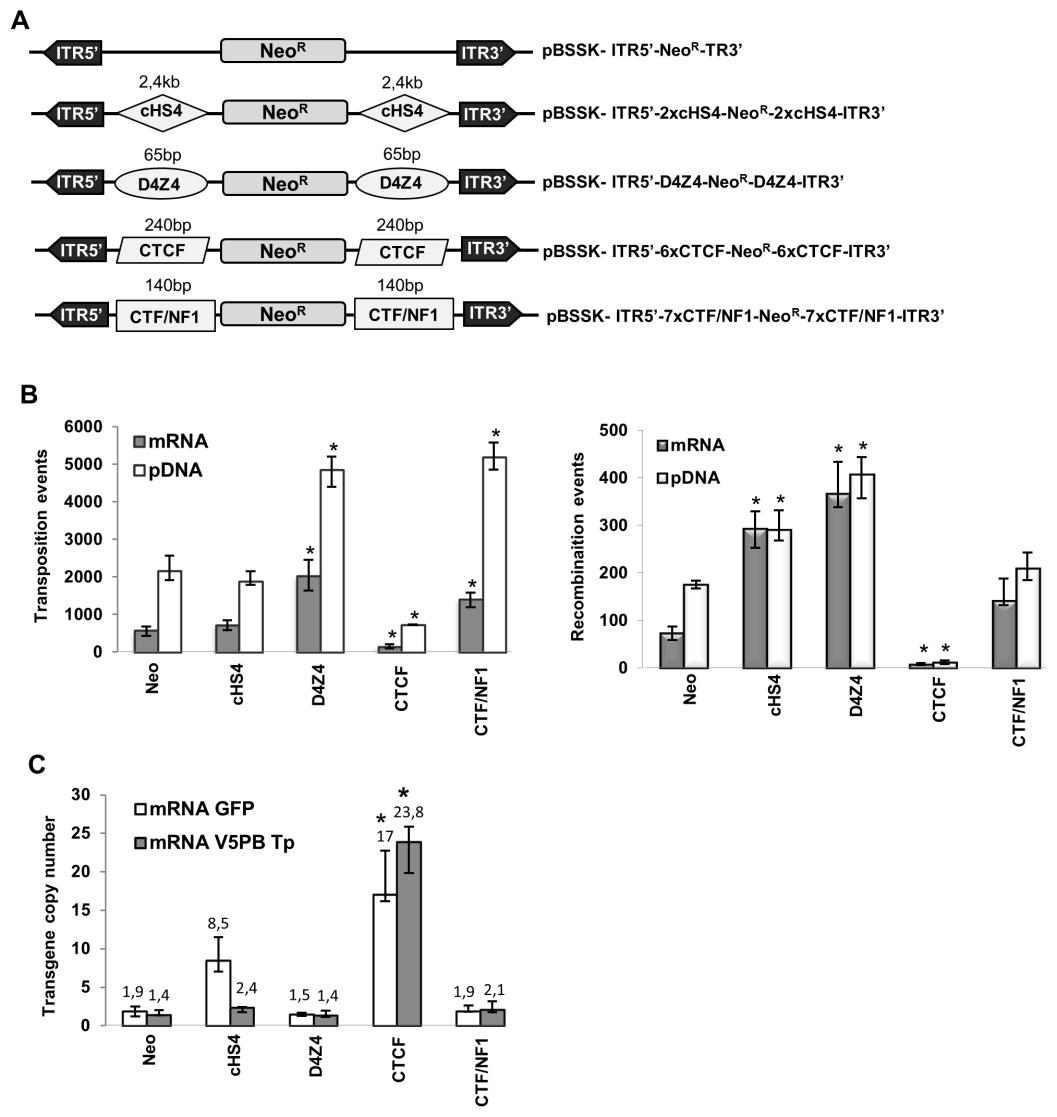
Effect of the insulating sequences. (A) Donor plasmids used for the insulator-effect study. The cHS4, D4Z4, CTCF or CTF/NF1 elements were cloned on either side of the Neo^R^ transgene. (B) Analysis of the different insulated vectors in *in* *vitro* colony forming assays in presence (left panel) or absence (right panel) of transposase. Cells were transfected with 187.5 ng of *PB* pDNA or mRNA plus 187.5 ng of each insulated donor plasmid (left panel). To consider only transposition events, the number of colonies observed in the presence of the transposase source was subtracted by the number of colonies obtained in the respective negative control performed without transposase for each quantity (right panel). Values represent the average of 3 experiments done in triplicate. * Indicates statistically significant differences between the non-insulated and the insulated Neo^R^ transposons (p<0.05). (C) Impact of the insulating sequences on the transgene copy number in pools of colonies. qPCR was performed on genomic DNA from pools of colonies with Neo^R^ specific primers. The mean copy number was calculated relative to that of the reference gene GAPDH in the human genome. * Indicates statistically significant differences between the non-insulated and the insulated Neo^R^ transposons (p<0.05). White bars correspond to plasmid transfection performed in the absence of the *piggyBac* transposase.

To prevent frequent post-integrative transcriptional silencing of the transgene, the transposable Neo^R^ cassette was bracketed with the cHS4, D4Z4, CTCF and CTF/NF1 insulators, and these constructs were assayed in transposition assays (Figure 5A). To find out whether insulators mediated position-effect protection and/or affected the transposition or spontaneous recombination rate, the transgene copy number was also estimated, since cells harboring silenced transgenes may not form colonies under antibiotic selection. All the constructs led to transposition events (Figure 5B, left panel). The cHS4 insulator gave similar colony and transgene copy numbers as the non-insulated control in the presence of transposase (Figure 5C). However, the recombination control showed a higher colony number (Figure 5B, right panel) and 4-fold more insertions. Therefore, cHS4 may have insulator effect when copies are integrated by recombination, which often led to tandem insertions, but to a lesser extent in the case of transposition (one copy per locus). Transposition assays performed using equimolar amounts of the non-insulated and cHS4 insulated donor vectors, to consider the larger size of the cHS4 plasmid, gave comparable results (Figure S4). In the presence of the *PB* pDNA or mRNA, the D4Z4 element respectively increased the number of colonies by 2- and 3.4-fold, and the CTF/NF1 by 2.5- and 2.4-fold. Since the D4Z4, CTF/NF1 and non-insulated Neo^R^ constructs showed equivalent Neo^R^ copy number with or without transposase, the increase in colony number was probably due to insulating protection rather than a more efficient transposition (or recombination). Thus, the D4Z4 and CTF/NF1 elements had insulator functions when combined with transposition. The CTCF reduced the number of colonies by 3-fold in the presence of transposase even though numerous transposons were integrated (>17 copies). This element was probably acting as a silencer by inhibiting the transgene transcription or by epigenetic silencing, rather than influencing transgene mobilization. 

## Discussion

Limiting or cutting-off transposase expression is a major challenge to enhance the biosafety of transposon-based gene delivery tools [30]. Several alternatives are investigated to control transposase expression in terms of quantity and duration: (i) inducible transposase expression [16], (ii) self-inactivating systems [31], (iii) delivery of a purified transposase [32]. However, these strategies are limited: pDNA-based systems pose the issue of transposase gene recombination in the cellular genome; active transposase proteins are not easy and expensive to produce. In this study, we focused on providing the transposase as an mRNA to control the transposase expression and ensure irreversible transgene integration. *In vitro*-transcribed mRNAs were successfully used with various transposon [20,21,33]. Here, a step-by-step comparison between *PB* mRNA and pDNA was carried out for the first time in terms of transposase expression, transposition efficiency and genotoxic effects in order to calibrate and secure the system.

The transfected *PB* mRNA led to a narrow window of expression and fewer transposition events due to its short half-life, compared to the mean half-life of endogenous human mRNAs (10 h, [34]), and that the proportion of mRNA potentially kept in cytoplasmic granules is not sufficient to allow prolonged protein expression [35]. Although theoretically impossible, a few cases of mRNA insertion through chimeric retropseudogenes have been reported [36], but their functionality has not been demonstrated. In our experiments, no integration was detected following *PB* mRNA transfection. In contrast, episomal pDNA and transposase gene integrations were detected following plasmid transfection, confirming that mRNA provides a decisive advantage in limiting transposase expression. Compared to other transposases [37] or related proteins [38], the *PB* transposase has a long half-life. As far as we are aware, this is the first time that a eukaryotic transposase mRNA and protein half-lives have been calculated, although these are essential milestones for monitoring transposase bioavailability and transposition events.

Neither small amounts of mRNA or pDNA transposase, nor transposition events caused major cell damage or cytotoxicity, indicating that the DNA damage cellular response was not overwhelmed [17]. Moreover, transposon systems were found less genotoxic than other non-viral integrative systems such as the ϕC31 integrase [39]. As reported earlier, our *PB* transposon integrated only few copies of the transgene thus, limiting insertional mutations [40]. Consequently, this transposon system appears to be safer than the other technologies and could be further optimized by mRNA engineering.

Once integrated, the gene of interest must be properly expressed, but newly integrated transgenes can be rapidly silenced by the host machinery [22]. A first strategy to circumvent silencing is to target high and continuous gene expression sites (reviewed in 41). A second solution is to use hyperactive transposases to increase the number of transgene insertions [17], but this also enhances the risk of insertional mutagenesis. The third solution is to use insulators to isolate the transgene from its surrounding environment. Insulators act as genetic barriers preventing deleterious heterochromatinization or activation of regulatory elements of the transgene but also of nearby cellular genes [42]. The insulated transgene is expected to behave as an autonomously-regulated expression unit [27].

In our hands, the cHS4 insulator did not improve the *PB* system. This insulator has been shown to be efficient when combined with transposon vectors, but cHS4 is also known not to provide absolute protection and to be functional in only certain cell types [25]. The transgene size effect could be ruled out since *PB* is able to efficiently mobilize large transposons [11]. The D4Z4 and CTF/NF1 minute elements led to a doubling of the colony number with no increase in the transgene copy number, suggesting a protective effect on transgene expression, as described previously [27,28]. This is consistent with other experiments performed with *Sleeping Beauty*, demonstrating that insulators did not affect pDNA transport or transposon mobilization, but only influenced transgene expression following integration into chromosomal chromatin, i.e. by preventing silencing effects and/or by directly activating the transcription of the transgene [25]. 

Overall, engineered transposase mRNA and insulated transposons could improve the quality of the transposition process and the quality of the transgene expression without increasing the number of inserted transgenes. It will be interesting to further assess the use of transposase mRNA and insulated transposons in the context of *ex vivo* gene or cell therapies, or the production of factory cells by generating genetically stable and efficient master cell banks. 

## Supporting Information

Table S1 **Antibodies used in these study.**
(PDF)

Table S2 **Names and sequences of the primers used in these study.**
(PDF)

Figure S1 **Control of *invitro* synthesized mRNA quality.** Quality and effective polyadenylation of mRNA (pA) were checked on 0,8% agarose gel electrophoresis. Before loading, mRNA was denatured during 10 min at 65°C.
(PDF)

Figure S2 **mRNA *piggyBac*-based transfection is efficient in various mammalian cell lines.** 1.10^5^ HEK 293 cells (upper panel) or CHO cells (lower panel) were transfected with 200 ng of V5PB mRNA alongside with a donor plasmid carrying the neomycin resistance gene (200 ng). After 15 days under antibiotic selection, resistant colonies were stained and counted to attest transposition efficiency. GFP mRNA (200 ng) served as a negative control (Mock=without transposase) corresponding to recombination events. Data are a representative image of 3 experiments done in triplicate.
(PDF)

Figure S3 **Cell proliferation assay after transfection and/or transposition assay.** 10^4^ cells were transfected with 187.5 ng of V5PB or GFP mRNA or pDNA, and/or with 187.5 ng of pBSK ITR-Neo^R^-ITR. GFP mRNA and pDNA were both used to exclude any impact of the gene sequence on the test results. Cell proliferation was assayed 24 h (dark grey bars) and 48 h (light grey bars) post-transfection by performing an MTT assay according to the Manufacturer’s instructions (CellTiter96^®^ Non-Radioactive Proliferation assay, Promega, Madison WI, USA). The cell propagation was calculated by subtracting the absorption at 650 nm (background absorbance) from the absorption at 595 nm (sample absorbance). The propagation of non-transfected cells was taken to be 100%. Values represent the mean ± SD (n=4). PEI: jetPEI only treated cells. No statistical difference in the signal was observed between the mock control and the other conditions using the Kruskal-Wallis test with p<0.05.
(PDF)

Figure S4 **Transposition assay using equimolar amount of the uninsulated and cHS4 insulated Neo plasmids.** To consider the large size of the cHS4 sequence and to transfect the same number of plasmid molecules, transposition assays were performed using 200 ng of pBSK ITR5’-Neo^R-^ITR3’ (black bars) or 400 ng of pBSK ITR5’-2xcHS4-Neo^R-2x^cHS4-ITR3’ (grey bars), alongside with 200 ng of the V5PB mRNA or GFP mRNA (negative control). Resistant colonies were stained with 70%EtOH-methylene blue and counted. The figure represents data of three experiments done in triplicate.
(PDF)
