# Supplementary material for: Optimization of the piggyBac Transposon Using mRNA and Insulators: Toward a More Reliable Gene Delivery System
Source: PLoS One. 2013 Dec 3;8(12):e82559. doi: 10.1371/journal.pone.0082559 (PMC3849487; doi:10.1371/journal.pone.0082559)
Supplement: Table S1 — Antibodies used in these study. (PDF) [file pone.0082559.s001.pdf]

Protein	Antibody
V5PB transposase	Mouse antiV5-HRP antibody (Invitrogen/Life Technologies)
Menin	Home-made
β-actin	Chicken polyclonal β-Actin antibody-Loading Control, Abcam, Cambridge, UK
γ-H2AX	-Primary rabbit anti-γ-H2AX antibody (Phospho-Histone H2A.X Ser139 Antibody, Cell Signaling Technology, Danvers MA, USA)<br>-Secondary mouse anti-rabbit IgG-HRP (Santa Cruz Biotechnology, Santa Cruz CA, USA)
