# Supplementary material for: Optimization of the piggyBac Transposon Using mRNA and Insulators: Toward a More Reliable Gene Delivery System
Source: PLoS One. 2013 Dec 3;8(12):e82559. doi: 10.1371/journal.pone.0082559 (PMC3849487; doi:10.1371/journal.pone.0082559)
Supplement: Table S2 — Names and sequences of the primers used in these study. (PDF) [file pone.0082559.s002.pdf]

Primer	Sequence	Program
V5PB- sense	5’ -cag caa gta cgg cat caa ga- 3’	40 cycles :<br><br>3 min at 94°C<br>30 s at 94°C<br>15 s at 55°C<br>30 s at 72°C
V5PB-antisense	5’ -gtc agc ttg tag ggc tcc tg- 3’	
18S-sense	5’ -gtg atg acc tgc agc aga aa- 3’	
18S-antisense	5’ -cag gtt ccg cat gaa ctt tt- 3’	
Neo-For	5’-ctg cat acg ctt gat ccg g-3’	
Neo-Rev	5’-atg ttt cgc ttg gtg gtc g-3’	
GAPDH-For	5’-ccc aaa gtc ctc ctg ttt ca-3’	
GAPDH-Rev	5’-gtt ccc agg act gga ctg tg-3’	
