# Supplementary figures and images for: Optimization of the piggyBac Transposon Using mRNA and Insulators: Toward a More Reliable Gene Delivery System
Source: PLoS One. 2013 Dec 3;8(12):e82559. doi: 10.1371/journal.pone.0082559 (PMC3849487; doi:10.1371/journal.pone.0082559)

M

V5PB

V5PB(pA)

GFP

GFP(pA)

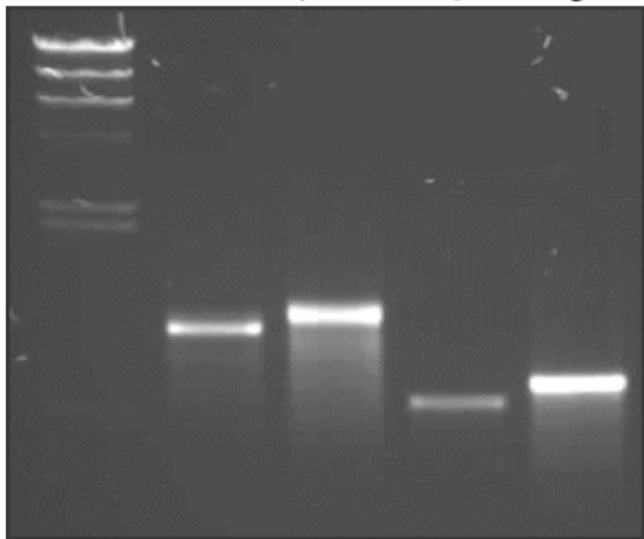

Supplement: Figure S1 — Control of invitro synthesized mRNA quality. Quality and effective polyadenylation of mRNA (pA) were checked on 0,8% agarose gel electrophoresis. Before loading, mRNA was denatured during 10 min at 65°C. (PDF) [file pone.0082559.s003.pdf]

**Mock**

**V5PB**

**HEK 293**

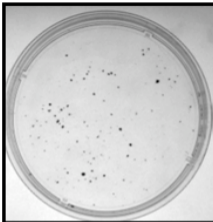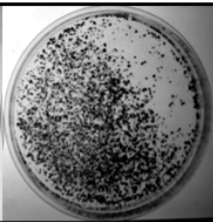

**CHO**

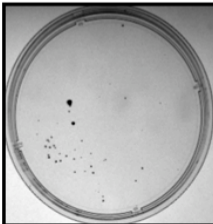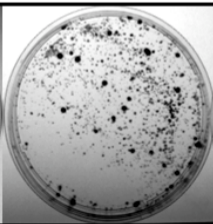

Supplement: Figure S2 — mRNA piggyBac-based transfection is efficient in various mammalian cell lines. 1.105 HEK 293 cells (upper panel) or CHO cells (lower panel) were transfected with 200 ng of V5PB mRNA alongside with a donor plasmid carrying the neomycin resistance gene (200 ng). After 15 days under antibiotic selection, resistant colonies were stained and counted to attest transposition efficiency. GFP mRNA (200 ng) served as a negative control (Mock=without transposase) corresponding to recombination events. Data are a representative image of 3 experiments done in triplicate. (PDF) [file pone.0082559.s004.pdf]

% of viable cells

mRNA

pDNA

Mock  
PEI  
GFP  
GFP+Neo  
V5PB  
V5PB+Neo  
GFP  
GFP+Neo  
V5PB  
V5PB+Neo  
Neo

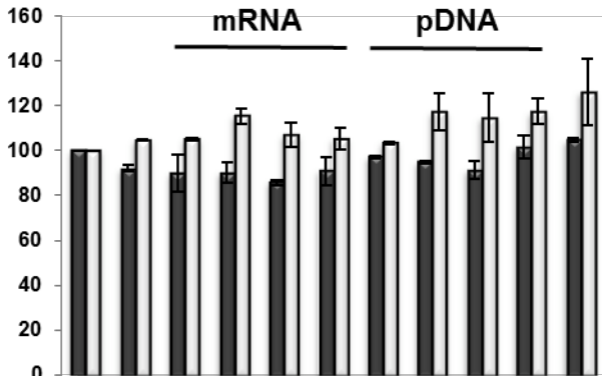

Supplement: Figure S3 — Cell proliferation assay after transfection and/or transposition assay. 104 cells were transfected with 187.5 ng of V5PB or GFP mRNA or pDNA, and/or with 187.5 ng of pBSK ITR-NeoR-ITR. GFP mRNA and pDNA were both used to exclude any impact of the gene sequence on the test results. Cell proliferation was assayed 24 h (dark grey bars) and 48 h (light grey bars) post-transfection by performing an MTT assay according to the Manufacturer’s instructions (CellTiter96® Non-Radioactive Proliferation assay, Promega, Madison WI, USA). The cell propagation was calculated by subtracting the absorption at 650 nm (background absorbance) from the absorption at 595 nm (sample absorbance). The propagation of non-transfected cells was taken to be 100%. Values represent the mean ± SD (n=4). PEI: jetPEI only treated cells. No statistical difference in the signal was observed between the mock control and the other conditions using the Kruskal-Wallis test with p<0.05. (PDF) [file pone.0082559.s005.pdf]

Number of colonies

■ Neo    ■ cHS4

GFP

V5PB

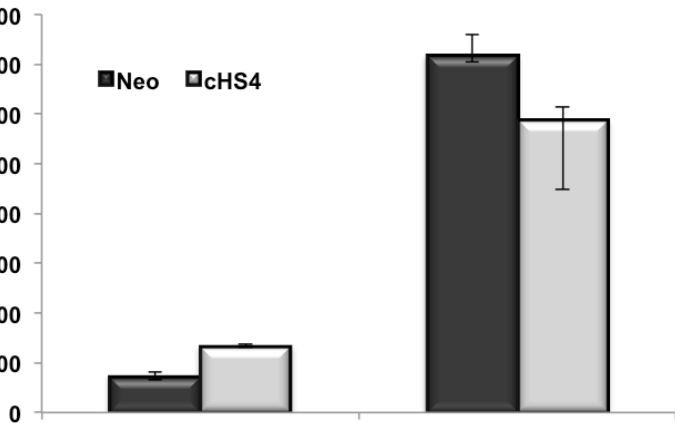

Supplement: Figure S4 — Transposition assay using equimolar amount of the uninsulated and cHS4 insulated Neo plasmids. To consider the large size of the cHS4 sequence and to transfect the same number of plasmid molecules, transposition assays were performed using 200 ng of pBSK ITR5’-NeoR-ITR3’ (black bars) or 400 ng of pBSK ITR5’-2xcHS4-NeoR-2xcHS4-ITR3’ (grey bars), alongside with 200 ng of the V5PB mRNA or GFP mRNA (negative control). Resistant colonies were stained with 70%EtOH-methylene blue and counted. The figure represents data of three experiments done in triplicate. (PDF) [file pone.0082559.s006.pdf]
